# A Limited Association of *OGG1* Ser326Cys Polymorphism for Adenocarcinoma of the Lung

**DOI:** 10.2188/jea.12.258

**Published:** 2007-11-30

**Authors:** Hidemi Ito, Nobuyuki Hamajima, Toshiro Takezaki, Keitaro Matsuo, Kazuo Tajima, Shunzo Hatooka, Tetsuya Mitsudomi, Motokazu Suyama, Shigeki Sato, Ryuzo Ueda

**Affiliations:** 1 Division of Epidemiology and Prevention, Aichi Cancer Center Research Institute.; 2 Department of Thoracic Surgery, Aichi Cancer Center Hospital.; 3 2^nd^ Department of Internal Medicine, Nagoya City University School of Medicine.; 4 Nagoya University Graduate School of Medicine.

**Keywords:** *OGG1* polymorphism, lung adenocarcinoma, PCR-CTPP

## Abstract

The 8-oxoguanine DNA glycosylase (OGG1) repairs DNA by removing 8-hydroxyguanine, a highly mutagenic oxidative DNA adduct. Recently, the gene for OGG1 was cloned and several polymorphisms have been reported. Because environmental carcinogens produce 8-hydroxyguanine residues that potentially cause oncogenic mutations by mismatching to this modified base, the capacity to repair these lesions can be involved in cancer susceptibility. This study investigated the association between *OGG1* Ser326Cys polymorphism and risk of the lung adenocarcinoma for Japanese by a prevalent case-control study in Japan. The subjects comprised 138 cases and 241 non-cancer outpatients as controls. *OGG1* gene polymorphism was genotyped by a PCR-CTPP (polymerase chain reaction with confronting two-pair primers) method. The distribution of *OGG1* Ser326Cys genotype among controls (*Ser/Ser*, 28.3%; *Ser/Cys*, 49.2%; and *Cys/Cys*, 22.5%) was not different from that among cases (*Ser/Ser*, 29.0%; *Ser/Cys*, 51.4%; and *Cys/Cys*, 24.0%). The sex-age adjusted odds ratio (OR) was 1.06 with 95% confidence interval (CI) 0.64-1.76 for *Ser/Cys* genotype and 0.81 with 0.44-1.52 for *Cys/Cys* genotype. The ORs according to the interval between diagnosis and study enrollment were also examined because the polymorphism was a potential prognostic factor of lung cancer. The ORs of *Ser/Cys* and *Cys/Cys* genotypes in the cases less than 3 years after diagnosis were higher than overall ORs; 1.86 (95%CI, 0.91 -3.77), and 1.46 (0.64-3.35), respectively. The OR for smoking was not statistically different among genotype, though the sample size was too small to detect even a moderate interaction. This study supported the first study by Sugimura *et al* (Cancer Epidemiol Biomarkers Prev, 1999; 8: 669-674), that the association of *OGG1* Ser326Cys polymorphism was limited for the risk of lung adenocarcinoma.

## INTRODUCTION

Oxidative DNA damage is thought to cause mutations, which can activate oncogenes or inactivate tumor suppresser genes finally leading to cancer^1^^,^^2^^)^. To protect the damage, organisms have developed highly efficient DNA repair machineries^3^^,^^4^^)^. Interindividual variation in the repair capacity has been implicated as a cancer susceptibility factor. 8-hydroxyguanine is one of the major forms of oxidative DNA damage produced by reactive oxygen species (ROS), and causes mutagenic / carcinogenic DNA misreading through G:C to T:A transversion^5^^,^^6^^)^. G:C to T:A transversion are widely seen in tumors *e. g.* in lung tumors where these transversion are very frequently found in the *p53* gene and compared with other tumors such as those of colon or breast^7^^,^^8^^)^. Several kinds of human cancer tissues, including lung cancer, showed higher levels of 8-hydroxyguanine compared with their non-cancerous counterparts^9^^-^^11^^)^. These findings indicate that this kind of oxidative DNA damage causes human cancer development.

The human 8-oxoguanine DNA glycosylase 1 is one of 8-hydroxyguanine repair enzymes^12^^-^^16^^)^. *OGG1* is the gene encoding the glycosylase, which belongs to the base excision repair gene family^17^^)^. Studies on genetic structure have revealed the presence of several polymorphisms within the *OGG1* locus^18^^,^^19^^)^. Among them, Ser326Cys polymorphism has been shown to be very common in Japanese and Chinese populations^19^^-^^21^^)^, but less common in Caucasian populations^20^^,^^22^^)^. The Cys326 protein has been shown to have about 7 times weaker 8-hydroxyguanine-repair capacity than Ser326 protein in complementation assay using *Escherichia coli* mutant strain deficient in 8-hydroxyguanine^19^^)^. However, Ser326Cys polymorphism did not correlate with the mean 8-hydroxyguanine levels in peripheral lymphocytes^19^^)^ and nor with the adduct levels in human lung samples^23^^)^. In addition, recent studies reported no difference in the catalytic activities between *Ser326* and *Cys326* alleles^24^^,^^25^^)^. Therefore, the difference in the function *in vivo* between the two alleles remains unclear.

The association between *OGG1* Ser326Cys polymorphism and the risk of lung cancer has been investigated in 3 studies^19^^,^^20^^,^^22^^)^. In all studies, the frequencies of *Cys/Cys* genotype were slightly higher among cases than among controls, but not significant. For other cancers, a significant association with the risk of esophageal cancer risk has been reported in China^21^^)^.

In Japan, lung cancer is the leading cause of deaths among malignancies^26^^)^. The cumulative incidence of lung adenocarcinoma has been steadily increasing for both males and females, while that for lung squamous cell carcinoma was almost constant from 1970s^27^^)^. The reason is not clear, but the change in the kind of cigarettes from high-tar no-filtered to low-tar filtered is suspected to be one of the factors^27^^)^. Although the association of old type cigarettes with the adenocarcinoma is weaker than with the squamous or small cell carcinoma, the adenocarcinoma may have a stronger association with low-tar filtered cigarettes. In order to detect high-risk individuals, studies on the interactions between genetic traits and smoking should be conducted for many tobacco-related polymorphisms. In this paper, we examined the association between Ser326Cys polymorphism and adenocarcinoma of the lung by a prevalent case-control study with hospital controls. Furthermore, the effect modification by this polymorphism was examined for smoking habit.

## MAERIALS AND METHODS

### Study subjects

Cases were lung cancer patients who visited Aichi Cancer center Hospital during 1999-2000. At the first stage, they were asked by their doctors in charge whether to participate in this study or not (not counted how many outpatients were asked by the doctors). At the second stage, the assentients at first stage were enrolled after written informed consent by staffs of Division of Epidemiology and Prevention. With a few exceptions all agree to participate in this study. A total of 138 Japanese patients with adenocarcinoma of the lung aged 26 to 80 years (68 males and 70 females) at diagnosis and 241 non-cancer controls aged 39-69 years (118 males and 123 females) were recruited. The cases had been diagnosed in the past 17 years at Aichi Cancer Center Hospital. The pathological distribution was as follows: 42 with well-differential adenocarcinoma, 73 with moderate-differential adenocarcinoma and 18 with poorly-differential adenocarcinoma. The controls were outpatients without a history of cancer who underwent gastroscopy, as described in our previous paper^28^^)^. All subjects gave written informed consent for polymorphism genotyping, completed a self-administered questionnaire and provided a 7 ml of peripheral blood sample. Controls included 97 (40.2% out of 241) participants stated to be under medication for 107 disease (not confirmed by their medical records); 23 with gastric/duodenal ulcer, another 23 for so-called gastritis, 16 with hypertension, 8 for pain including arthritis and lumbargo, 7 with diabetes mellitus, 7 with hyperlipidemia, 3 with ischemic heart disease, 3 with thyroid disease, 2 with gynecological disease, 2 with hyperuricemia, 2 with Meniere disease, 2 with prostate disease, 1 with ulcerative colitis, 1 with pancreatitis, 1 with asthma, 1 with arrhythmia, 1 for epilepsy, 1 for neurosis, 1 with liver cirrhosis, 1 for ulticaria, and 1 after cerebral infarction. Smoking status at diagnosis for cases or at interview for controls was classified into three categories; current smokers including ex-smokers within one year after the cessation, never smokers including individuals who smoked less than 100 cigarettes in their lifetime, and former smokers for the rest who quitted smoking. The Institutional Review Board of Aichi Cancer Center approved this study before the study started.

### Genotyping procedure

**Figure 1.**
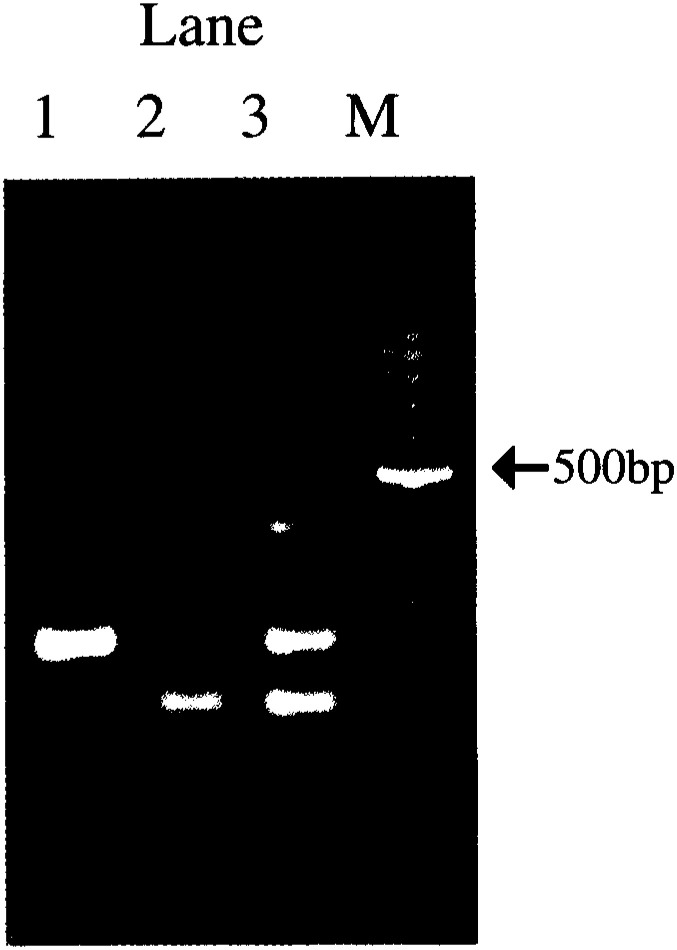
Representative results for the *OGG1* Ser326Cys polymorphism by PCR-CTPP method. DNA fragments stained with ethidium bromide are shown. Lane 1 for *Ser/Ser*, lane 2 for *Ser/Cys*, lane 3 for *Cys/Cys*, and lane M for a 100-bp DNA ladder.

DNA was extracted from 200 *μ*l buffy coat reserved -40°C by a QIAamp Blood Mini Kit (Qiagen, Valencia, CA) and *OGG1* Ser326Cys (78435 C to G substitution) polymorphism was genotyped by a PCR-CTPP (polymerase chain reaction with confronting two-pair primers) method developed independently in our laboratory^29^^)^, which is a similar method by Liu et al^30^^)^. Each 25 *μ*l reaction tube contained 30-100ng DNA, 0.18mM dNTPs, 12.5pmol each primer, 0.5 U AmpliTaq Gold (Perkin-Elmer, Foster City, CA) and 2.5 *μ*l of 10 x PCR buffer including 15mM MgCl_2_. The four primers were F1, 5′-CAG CCC AGA CCC AGT GGA CTC-3′; R1, 5′-TGG CTC CTG AGC ATG GCG GG-3′; F2, 5′-CAG TGC CGA CCT GCG CCA ATG-3′; and R2, 5′-GGT AGT CAC AGG GAG GCC CC. Primer pair F1 & R1 for the *C* allele (*Ser326*) and F2 & R2 for the *G* allele (*326Cys*) produced allele-specific bands of 252bp and 194bp, respectively, as well as a common 406bp band between F1 and R2. The PCR was conducted as follows; 10 min of initial denaturation at 95°C, followed by 30 cycles of 1 min at 95°C, 1 min at 64°C and 1 min at 72°C, then 5 min extension at 72°C. Figure 1 shows the representative result of genotyping by the PCR-CTPP method.

### Statistical Analysis

All statistical analyses were performed using STATA v.7.0 software (STATA, College Station, TX). Accordance with Hardy-Weinberg equilibrium, which indicates an absence of discrepancies between genotype and allele frequencies, was examined for controls with a *χ*^2^ test. Crude and sex-age adjusted odds ratios (ORs) and 95% confidence intervals (CIs) were calculated by an unconditional logistic regression model. Gene-environment interactions between *OGG1* polymorphism and smoking habit was also estimated by the logistic model, which included an interaction term as well as variables for exposure (smoking), genotype (*OGG1*) and potential confounders (age and sex).

## RESULTS

The characteristics among the cases and controls are shown in Table 1. The mean age was 60.7 years for cases and 56.8 years for controls. The median interval from diagnosis to study enrollment was 3.2 years, and the cases diagnosed in the past 2 years were 71 (51.4%). There was no difference in the percentage of smokers between cases (23.2%) and controls (22.8%), but the cases tended to be heavier smokers than controls, as shown in Table 1.

**Table 1.** Characteristics of cases and controls.

Characteristics		Cases (%)	Controls (%)
		(n=138)	(n=241)
Sex	Male	68 (49.3)	118 (49.0)
	Female	70 (50.7)	123 (51.0)
Age at diagnosis/interview	<= 50	15 (10.9)	46 (19.1)
	51-60	43 (31.2)	90 (37.3)
	61-70	54 (39.1)	105 (43.6)
	>= 71	26 (18.8)	0 ( 0.0)
	Mean age ± SD	60.7 ± 9.4	56.8 ±7.9
The intervals from diagnosis	< 3 years	71 (51.4)	
	>= 3 years	67 (48.6)	
Smoking status	Never smokers	78 (56.5)	140 (58.1)
	Former smokers	28 (20.3)	46 (19.1)
	Current smokers	32 (23.2)	55 (22.8)
	Cigarette-years< 800	7 ( 8.8)	26 (11.6)
	Cigarette-years>= 800	25 (18.1)	27 (11.2)
	Unknown	0 ( 0.0)	2 ( 0.8)

The allele frequencies of *OGG1 Ser326* for controls and cases were 0.55 and 0.53, respectively. The genotype was 28.3% for *Ser/Ser*, 49.2% for *Ser/Cys* and 22.5% for *Cys/Cys* genotype among controls (Table 2). The distribution of genotype among controls was in accordance with Hardy-Weinberg equilibrium (*χ*^2^=0.043, p=0.837). The distribution among cases did not differ from that among controls: 29.0% for *Ser/Ser* and 51.4% for *Ser/Cys* and 19.6% for *Cys/Cys*. There was a significant difference in the distribution of genotypes between the cases enrolled less than 3 years after diagnosis (recent cases) and those enrolled 3 years or longer after diagnosis (longer survivors): 19.7% for *Ser/Ser*, 56.3% for *Ser/Cys*, and 24.0% for *Cys/Cys* among the recent cases; 38.8%, 46.3%, and 14.9% among the longer survivors, respectively (Table 2).

**Table 2.** Distribution of genotype among cases and controls.

Genotype	No. of cases (%)			No. of controls (%) ^†^

	Total	The interval from diagnosis		

		< 3 years*	>= 3 years*	
*Ser/Ser*	40 (29.0)	14 (19.7)	26 (38.8)	68 (28.3)
*Ser/Cys*	71 (51.4)	40 (56.3)	31 (46.3)	118 (49.2)
*Cys/Cys*	27 (19.6)	17 (24.0)	10 (14.9)	54 (22.5)

* Genotype distribution was significantly different; *χ*^2^ (2)= 6.45 and p=0.040.

^†^ One subject could not genotyped.

As shown in Table 3, the crude and adjusted ORs for *Ser/Cys* or *Cys/Cys* genotypes compared with *Ser/Ser* were not significant. We also analyzed the ORs by the interval from diagnosis, because there was a significant difference in *OGG1* genotype distribution between the recent cases and the longer survivors. The adjusted ORs compared with *Ser/Ser* genotype were 1.86 (95% CI, 0.83-3.24) for *Ser/Cys* and 1.53 (0.69-3.38) for *Cys/Cys* in the recent cases, and 0.69 (0.38-1.25) and 0.48 (0.22-1.09) in the longer survivors, respectively. The adjusted ORs for *Ser/Cys* and *Cys/Cys* genotypes combined were 1.72 (0.88-3.39) in the recent cases and 0.64 (0.36-1.14) in the longer survivors. The OR of the genotype among current smokers did not differ markedly from that among never smokers, though former smokers had a lower OR (Table 4).

**Table 3.** The overall ORs and that according to the interval from diagnosis.

	Genotype	Crude		Adjusted*	
	
		OR	95%CI	OR	95%CI
Total	*Ser/Ser*	1.00		1.00	
	*Ser/Cys*	1.02	0.63-1.67	1.06	0.64-1.76
	*Cys/Cys*	0.85	0.46-1.56	0.81	0.44-1.52
The interval from diagnosis					
< 3 years	*Ser/Ser*	1.00		1.00	
	*Ser/Cys*	1.64	0.83-3.24	1.86	0.91-3.77
	*Cys/Cys*	1.53	0.69-3.38	1.46	0.64-3.35
>= 3 years	*Ser/Ser*	1.00		1.00	
	*Ser/Cys*	0.69	0.38-1.25	0.71	0.39-1.31
	*Cys/Cys*	0.48	0.22-1.09	0.48	0.54-1.64

*Adjusted for age and sex.

**Table 4.** Adjusted ORs according to smoking habit.

Subjects	Genotype	No. of cases/controls	aOR*	95%CI
Total				
Never smokers	*Ser/Ser*	23/45	1.00	
	*Ser/Cys*	41/65	1.36	0.71-2.62
	*Cys/Cys*	14/30	1.00	0.46-2.36
Former smokers	*Ser/Ser*	8/11	1.00	
	*Ser/Cys*	12/20	0.56	0.15-2.06
	*Cys/Cys*	8/14	0.35	0.08-1.60
Current smokers	*Ser/Ser*	9/12	1.00	
	*Ser/Cys*	18/33	0.76	0.25-1.63
	*Cys/Cys*	5/10	0.87	0.27-2.76

*Adjusted for age and sex.

The age-sex-adjusted OR relative to never smokers was slightly high for former smokers and current smokers 1.18 (0.59-2.34) and 1.29 (0.67-2.49), respectively (Table 5). The ORs for smoking status according to the *OGG1* genotype show that the OR for current smokers was slightly high among individuals with *Cys/Cys* genotype (2.47, 0.50-12.24), though the interaction was not statistically significant.

**Table 5.** The ORs of smoking habit according to *OGG1* Ser326Cys genotype.

	No. of cases/controls	Adjusted for age and sex	

		OR	95%CI
Total			
Never smokers	78/140	1.00	
Former smokers	28/46	1.18	0.59-2.34
Current smokers	32/55	1.29	0.67-2.49
*Ser/Ser*			
Never smokers	23/45	1.00	
Former smokers	8/11	2.07	0.40-10.6
Current smokers	9/12	1.89	0.47-7.68
*Ser/Cys*			
Never smokers	41/65	1.00	
Former smokers	12/20	0.84	0.31-2.26
Current smokers	18/33	0.92	0.38-2.25
*Cys/Cys*			
Never smokers	14/30	1.00	
Former smokers	8/14	1.54	0.42-5.76
Current smokers	5/10	2.47	0.50-12.24

*Adjusted for age and sex.

## DISCUSSION

This was the first study that OGG1 Ser326Cys polymorphism was genotyped by the PCR-CTPP method^29^^)^, whose genotyping was quite clear as demonstrated in Figure 1. The method is easy, timesaving and inexpensive compared with PCR-SSCP. The genotype distribution was in accordance with Hardy-Weinberg law of equilibrium among controls (p=0.873), which partly supported appropriate control selection and correct genotyping. The genotype distribution (28.3% for *Ser/Ser*, 49.2% for *Ser/Cys*, and 22.5% for *Cys/Cys*) was similar to those previously reported by Sugimura *et al.* in Tokyo (27.7%, 57.4%, and 14.9%, respectively, n=94, p for Hardy-Weinberg equilibrium=0.103) and Okinawa populations (32.0%, 54.3%, and 13.7%, respectively, n=197, p for Hardy-Weinberg equilibrium=0.082)^22^^)^, though the proportion for *Cys/Cys* was relatively high. The distribution of the Ser326Cys polymorphism was quite different among ethnic groups. The frequency of *Cys326* allele in Japanese and Chinese populations was prevalent compared with other ethnic groups; *Cys/Cys* genotype was reported 3.6% for Australian Caucasian (n=138), 2.7% for Hungarian (n=149)^22^^)^ and 1.9% for Caucasian in Germany (n=105)^24^^)^.

In our study, compared with *Ser/Ser* genotype, the *Ser/Cys* and *Cys/Cys* genotypes were not significantly associated with the risk of adenocarcinoma of the lung. Because this was a prevalent case-control study, the estimated ORs indicated not only with disease occurrence but also with disease prognosis. In prevalent case-control studies, longer survivors are more likely to be sampled as cases^31^^)^. Accordingly, the factors related to shorter survival are less frequent among prevalent cases than among incident cases, which include those with poor prognosis as well as long survivors. It produces a lower OR for the prevalent cases than that estimated for the incidence cases. We divided the case subjects into 2 groups (less than 3 years after diagnosis versus 3 years or longer) and estimated the OR according to the interval from diagnosis. Although the difference was not statistically significant, the ORs of *Ser/Cys* and *Cys/Cys* genotypes compared with *Ser/Ser* genotype were larger in the recent cases than in the longer survivors. This finding implies that *OGG1* may influence prognosis of lung adenocarcinoma. To date, there have been no studies on the association between this polymorphism and cancer prognosis. The suspected prognostic effect should be examined prospectively in survival analysis adjusting for other prognostic factors.

In this study population, 70 (50.7%) of 138 patients with adenocarcinoma were women, and this proportion was similar to that for the whole patients with lung adenocarcinoma diagnosed at Aichi Cancer Center Hospital (46.8% of 828 patients between 1984 and 2000). The corresponding proportion was higher at Aichi Cancer Center than that reported in Japan^32^^)^. Therefore, the high proportion of women with adenocarcinoma in this study was reflected in the sex distribution at Aichi Cancer Center Hospital.

In the study by Sugimura et al.^22^^)^, the significant association between the *Cys/Cys* genotype of *OGG1* and lung cancer risk was observed only for squamous cell carcinoma (n=l 18), and a slightly elevated insignificant OR for adenocarcinoma of the lung (n=78) was also documented. Confirming the findings for the adenocarcinoma was required. Our finding for the recent cases was very similar in the size of OR to their results. Our study was also too short to confirm the association, but added information to the finding observed by the previous study. Although different histological types of lung adenocarcinoma may be associated differently with this polymorphism, neither this study nor previous studies were large enough to examine the possible heterogeneous association.

Biological evidence supporting the association with lung carcinoma risk is still limited. Previous reports showed that loss of hetrozygosity (LOH) of 3p, which includes *OGG1* gene, was very common in lung cancer tissues^21^^,^^24^^,^^25^^)^. Although mean 8-hydroxyguanine levels in lung tissue was higher with LOH than without LOH, the genotype of *OGG1* Ser326Cys was not associated with the level, but a polymorphism of *glutathione peroxidase I* gene also located in 3p was correlated^33^^)^. Recently an association between lung adenocarcinoma risk and new *OGG1* polymorphisms at exon 1 has been reported for Japanese^20^^)^. Further biological studies on these *OGG1* polymorphisms, especially in relation to environmental exposures, will be required to elucidate the biological roles in the carcinogenesis.

Currently, adenocarcinoma of the lung is considered to be associated with smoking, though the relative risk is lower than of squamous cell and small cell carcinoma^34^^-^^36^^)^. Since high levels of 8-hydroxyguanine have been detected in smokers’ lung tissue and leukocytes^37^^)^, the interaction with the *OGG1* genotype was to be examined. In our study, the OR of the genotype was not different between never smokers and current smokers, and the difference in the OR of smoking was not large among the genotypes; 1.89 for *Ser/Ser*, 0.92 for *Ser/Cys*, and 2.47 for *Cys/Cys*. This suggests that *OGG1* Ser326Cys polymorphism-smoking interaction was not marked in adenocarcinoma of the lung enough to be detected in our study. In the past studies, the possible interaction with smoking was reported for *CYP1A1* and *GSTM1*^38^^,^^39^^)^. Our previous study found the interaction between *L-myc* *L/S* polymorphism and smoking for esophageal cancer^40^^)^.

In conclusion, this study suggested that the association between *OGG1* Ser326Cys polymorphism and the risk of lung adenocarcinoma was limited, if any, but that the *Cys/Cys* genotype might be associated with the poor prognosis. In this study, no difference in the OR of smoking among the genotypes was observed. The sample size was not enough for detailed analysis, but this was the largest study on the association with lung adenocarcinoma so far.

## References

[1] Fearon ER. Human cancer syndromes: clues to the origin and nature of cancer. Science, 1997; 278: 1043-1050. 9353177 10.1126/science.278.5340.1043

[2] Mamett LJ. Oxyradicals and DNA damage. Carcinogenesis, 2000; 21: 361-370. 10688856 10.1093/carcin/21.3.361

[3] Laval J, Jurado J, Saprabaev M, Sidorkina O. Antimutagenic role of base-excision repair enzymes upon free radical-induced DNA damage. Mutat Res, 1998; 402: 93-102. 9675252 10.1016/s0027-5107(97)00286-8

[4] Bohr VA, Dianov GL. Oxidative DNA damage processing in nuclear and mitochondrial DNA. Biochimie, 1999; 81: 155-160. 10214920 10.1016/s0300-9084(99)80048-0

[5] Shibutani S, Takeshita M, Grollman AP. Insertion of specific bases during DNA synthesis past the oxidation-damaged base 8-oxodG. Nature, 1991; 349: 431-434. 1992344 10.1038/349431a0

[6] Cheng KC, Cahill DS, Kasai H, Nishimura S, Loeb LA. 8-Hydroxyguanine, an abundant form of oxidative DNA damage, causes G-T and A-C substitutions. J Biol Chem, 1992; 267: 166-172. 1730583

[7] Hussain SP, Harris CC. Molecular epidemiology of human cancer: contribution of mutation spectra studies of tumor suppressor genes. Cancer Res, 1998; 58: 4023-4037. 9751603

[8] Hollstein M, Sidransky D, Vogelstein B, Harris CC. p53 mutations in human cancers. Science, 1991; 253: 49-53. 1905840 10.1126/science.1905840

[9] Jaruga P, Zastawny TH, Skokowski J, Dizdaroglu M, Olinski R. Oxidative DNA base damage and antioxidant enzyme activities in human lung cancer. FEBS Lett, 1994; 341: 59-64. 8137923 10.1016/0014-5793(94)80240-8

[10] Kondo S, Toyokuni S, Iwasa Y, . Persistent oxidative stress in human colorectal carcinoma, but not on adenoma. Free Radic Biol Med, 1999; 27: 401-410. 10468215 10.1016/s0891-5849(99)00087-8

[11] Musarrat J, Arezina-Wilson J, Wani AA. Prognostic and aetiological relevance of 8-hydroxyguanosine in human breast carcinogenesis. Eur J Cancer, 1996; 32A: 1209-1214. 8758255 10.1016/0959-8049(96)00031-7

[12] van der Kemp PA, Thomas D, Barbey R, de Oliveira R, Boiteux S. Cloning and expression in *Escherichia coli* of the *OGG1* gene of *Saccharomyces cerevisiae*, which codes for a DNA glycosylase that excises 7,8-dihydro-8-oxoguanine and 2,6-diamino-4-hydroxy-5-*N*-methylformamidopyrimidine. Proc Natl Acad Sci U S A, 1996; 93: 5197-5202. 8643552 10.1073/pnas.93.11.5197 PMC39221

[13] Nash HM, Bruner SD, Scharer OD, . Cloning of a yeast 8-oxoguanine DNA glycosylase reveals the existence of a base-excision DNA-repair protein superfamily. Curr Biol, 1996; 6: 968-980. 8805338 10.1016/s0960-9822(02)00641-3

[14] Aburatani H, Hippo Y, Ishida T, . Cloning and characterization of mammalian 8-hydroxyguanine-specific DNA glycosylase/apurinic, apyrimidinic lyase, a functional *mut*M homologue. Cancer Res, 1997; 57: 2151-2156. 9187114

[15] Arai K, Morishita K, Shinmura K, . Cloning of a human homolog of the yeast *OGG1* gene that is involved in the repair of oxidative DNA damage. Oncogene, 1997; 14: 2857-2861. 9190902 10.1038/sj.onc.1201139

[16] Sakumi K, Furuichi M, Tsuzuki T, . Cloning and expression of cDNA for a human enzyme that hydrolyzes 8-oxo-dGTP, a mutagenic substrate for DNA synthesis. J Biol Chem, 1993; 268: 23524-23530. 8226881

[17] Dianov G, Bischoff C, Piotrowski J, Bohr VA. Repair pathways for processing of 8-oxoguanine in DNA by mammalian cell extracts. J Biol Chem, 1998; 273: 33811-33816. 9837971 10.1074/jbc.273.50.33811

[18] Ishida T, Takashima R, Fukayama M, . New DNA polymorphisms of human *MMH/OGG1* gene: prevalence of one polymorphism among lung-adenocarcinoma patients in Japanese. Int J Cancer, 1999; 80: 18-21. 9935223 10.1002/(sici)1097-0215(19990105)80:1<18::aid-ijc4>3.0.co;2-e

[19] Kohno T, Shimura K, Tosaka M, . Genetic polymorphisms and alternative splicing of the *hOGG1* gene, that is involved in the repair of 8-hydroxyguanine in damaged DNA. Oncogene, 1998; 16: 3219-3225. 9681819 10.1038/sj.onc.1201872

[20] Sugimura H, Kohno T, Wakai K, . *hOGG1* Ser326Cys polymorphism and lung cancer susceptibility. Cancer Epidemiol Biomarkers Prev, 1999; 8: 669-674. 10744126

[21] Xing DY, Tan W, Song N, Lin DX. Ser326Cys polymorphism in *hOGG1* gene and risk of esophageal cancer in a Chinese population. Int J Cancer, 2001; 95: 140-143. 11307145 10.1002/1097-0215(20010520)95:3<140::aid-ijc1024>3.0.co;2-2

[22] Wikman H, Risch A, Klimel F, . *hOGG1* polymorphism and loss of heterozygosity (LOH): significance for lung cancer susceptibility in a Caucasian population. Int J Cancer, 2000; 88: 932-937. 11093817 10.1002/1097-0215(20001215)88:6<932::aid-ijc15>3.0.co;2-p

[23] Hardie LJ, Briggs JA, Davidson LA, . The effect of hOGG1 and glutathione peroxidase I genotypes and 3p chromosomal loss on 8-hidroxydeoxyguanosine levels in lung cancer. Carcinogenesis, 2000; 21: 167-172. 10657953 10.1093/carcin/21.2.167

[24] Dherin C, Radicella JP, Dizdaroglu M, Boiteux S. Excision of oxidatively damaged DNA bases by the human alpha-hOgg1 protein and the polymorphic alpha-hOgg1 (Ser326Cys) protein which is frequently found in human populations. Nucleic Acids Res, 1999; 27: 4001-4007. 10497264 10.1093/nar/27.20.4001 PMC148667

[25] Janssen K, Schlink K, Gotte W, . DNA repair activity of 8-oxoguanine DNA glycosylase 1 (OGG1) in human lymphocytes is not dependent on genetic polymorphism Ser326/Cys326. Mutat Res, 2001; 486: 207-216. 11459633 10.1016/s0921-8777(01)00096-9

[26] Statistics and Information Department, Ministry of Health and Welfare. Vital statistics of Japan 1998, Vol 3, 2000. Health and Welfare Statistics Association, Tokyo, (in Japanese)

[27] Sobue T, Ajiki W, Tsukuma H, . Trends of lung cancer incidence by histologic type: a population-based study in Osaka, Japan. Jpn J Cancer Res, 1999; 90: 6-15. 10076559 10.1111/j.1349-7006.1999.tb00659.x PMC5925973

[28] Hamajima N, Matsuo K, Saito T, . Interleukin 1 polymorphisms, lifestyle factors, and *Helicobacter pylori* infection. Jpn J Cancer Res, 2001; 92: 383-389. 11346459 10.1111/j.1349-7006.2001.tb01106.x PMC5926728

[29] Hamajima N, Saito H, Matsuo K, Kozaki K, Takahashi T, Tajima K. Polymerase chain reaction with confronting two-pair primers for polymorphism genotyping. Jpn J Cancer Res., 2000; 91: 865-868. 11011111 10.1111/j.1349-7006.2000.tb01026.x PMC5926438

[30] Liu Q, Thorland EC, Heit JA, Sommer SS. Overlapping PCR for bidirectional PCR amplification of specific alleles: a rapid one-tube method for simultaneously differentiating homozygotes and heterozygotes. Genome Res, 1997; 7: 389-398. 9110178 10.1101/gr.7.4.389 PMC139149

[31] Hamajima N, Matsuo K, Yuasa H. Adjustment of prognostic effects in prevalent case-control studies on genotype. J Epidemiol, 2001; 11: 204-210, Corrections, 228. 11579927 10.2188/jea.11.204 PMC11769785

[32] Sekine I, Nagai K, Tsugane S, . Association between smoking and tumor progression in Japanese women with adenocarcinoma of the lung. Jpn J Cancer Res, 1999; 90: 129-135. 10189882 10.1111/j.1349-7006.1999.tb00725.x PMC5926042

[33] Hibi K, Takahashi T, Yamakawa K, . Three distinct regions involved in 3p deletion in human lung cancer. Oncogene, 1992; 7: 445-449. 1347916

[34] Asami S, Hirano T, Yamaguchi R, . Increase of a type of oxidative DNA damage, 8-hydroxyguanine, and its repair activity in human leukocytes by cigarette smoking. Cancer Res, 1996; 56: 2546-2549. 8653695

[35] Barbone F, Bovenzi M, Cavalleri F, Stanta G. Cigarette smoking and histologic type of lung cancer in men. Chest, 1997; 112: 1474-1479. 9404741 10.1378/chest.112.6.1474

[36] Kabet GC. Aspects of the epidemiology of the lung cancer in smokers and nonsmokers in the United States. Lung Cancer, 1996; 15: 1-20. 8865119 10.1016/0169-5002(95)00566-8

[37] Wei Q, Cheng L, Hong WK, Spitz MR. Reduced DNA repair capacity in lung cancer patients. Cancer Res, 1996; 56: 4103-4107. 8797573

[38] Nakachi K, Hayashi S, Kawajiri K, Imai K. Association of cigarette smoking and CYP1A1 polymorphisms with adenocarcinoma of the lung by grades of differentiation. Carcinogenesis, 1995; 16: 2209-2213 7554077 10.1093/carcin/16.9.2209

[39] Kawajiri K, Eguchi H, Nakachi K, Sekiya T, Yamamoto M. Association of CYP1A1 germ line polymorphisms with mutations of the p53 gene in lung cancer. Cancer Res, 1996; 56: 72-76 8548778

[40] Kumimoto H, Hamajima N, Nishizawa, . Different susceptibility of each *L-myc* genotype to esophageal cancer risk factors. Jpn J Cancer Res, 2001; 92: 735-739. 11473723 10.1111/j.1349-7006.2001.tb01155.x PMC5926774

